# Supplementary material for: Understandings of community participation and empowerment in primary health care in Emilia-Romagna, Italy: A qualitative interview study with practitioners and stakeholders
Source: PLoS One. 2024 Sep 26;19(9):e0310137. doi: 10.1371/journal.pone.0310137 (PMC11426527; doi:10.1371/journal.pone.0310137)
Supplement: S1 File — (PDF) [file pone.0310137.s002.pdf]

Interview guideline

Thank you for participating in this study. In this study we want to analyse from different perspectives how and which community-oriented approaches (e. g. group activities, Community Labs, CdS-boards) are promoted by the Case della Salute.

We are interested in how people in the community are involved, for example, in the knowledge and discussion of community needs and health resources, how their views are taken into account and whether community members have a voice regarding Case della Salute issues and Case della Salute activities.

We are also interested in identifying past, recent and future developments in this field, your general perception of this topic and the barriers and possibilities of these approaches in the context of Case della Salute. Furthermore, we would like to understand what factors/aspects hinder and favour their promotion.

We will conduct interviews on the topic of community participation and community empowerment with different actors: actors at national, regional, municipal level, people at the level of management of the Case della Salute, doing research, professionals who are involved in the implementation of community-oriented work and citizens representatives from third sector organisations.

If there are any questions you do not want or cannot answer, please let me know. In this interview we are interested in your personal experiences and your point of view, there is no right or wrong answer.

Do you have any questions so far?

1. Presentation and reference to the Case della Salute

First, could you please introduce yourself?

a. What is your professional background?

b. What is your professional position and what are your tasks?

c. To what extent do you deal with Case della Salute?

2. Developments of the Case della Salute

a. When looking back in time since you first became interested in the Case della Salute, what do you think are some **interesting developments** or **important milestones** in the Case della Salute or in the Case della Salute that you know?
   - In general
   - From the point of view of community participation and community empowerment

3. General understanding of the terms community participation and community empowerment
Thinking in a general way when you think of community participation and community empowerment:

a. What, in your opinion, **characterises community participation**? Can you give me an example so that I can imagine it better?
b. What, in your opinion, are the **generally important aspects** for **successful or functioning** community participation?

And also for 'community empowerment':

c. In your experience or opinion, what **characterises community empowerment**? Could you also give me an example here or describe a situation?

d. In your opinion, what **aspects** are **generally important** for **successful or functioning** community empowerment?

4. General evaluation of community participation and community empowerment in the Case della Salute

a. In your opinion, what **relevance** or **importance** do community participation and community empowerment have in the context of the Case della Salute?

b. What **consequences or results** do you expect from these approaches? (e.g., benefits)

c. How is the **importance** of doing community participation and community empowerment **perceived** by the people in the Case della Salute who are involved?

d. The **transfer of national or regional directives or guidelines to practice** is often a delicate point. Could you tell me something about how this transfer has been done so far with regard to the issues of community participation and community empowerment in the Case della Salute?

a. Where do barriers show up, in this moment, in the **transfer** to practice?
b. Where do you see opportunities for **transfer** to practice instead?

e. What can decision-makers/legislators learn **from practice** instead?

5. <u>Community participation and community empowerment approaches in the Case della Salute</u>

If stakeholder does not know the approaches/projects in detail:

Could you give me examples from the Case della Salute you know of, where community participation and/or community empowerment approaches/activities have been promoted?

- Could you list them?

If stakeholder knows of practical examples or has participated in them, ask in detail:

I now ask you to tell me about the community participation and/or community empowerment approaches/activities in which you have been or are participating.

- Could you first list the projects in which you are or were involved and then tell me about them in detail?

<u>Details of the different community-oriented approaches</u><br><br>**Now I would like to ask you to please tell us what exactly was done in this approach. I am interested from <u>beginning to end</u> in how the activity was carried out:**<br><br>a. First of all, I am interested in understanding, how did this approach/activity/project come about?<br><br>b. How did you come to participate in this activity?<br><br>c. What was your role, what were your tasks?<br><br><u>Goals of the community approach</u><br><br>d. Why was **this approach** chosen **for this community** (community needs, needs assessment, 'externally initiated')?<br><br>e. What **ideas** and perhaps **objectives** did you have for this approach?<br><br><u>Implementation of activities</u><br><br>f. How was this approach/activity/project **implemented**?<br><br>g. How were the individual meetings **organised**?

h. What **exactly happened** in the meetings?

Actors and target groups involved

i. Who were all the **actors involved** and what were their tasks?

j. How can I imagine the **collaboration** of the actors involved? Could you give me an example illustrating how the exchange worked?

k. **Who should have been involved** in this approach? Who did you **want to involve**?

l. Who was **actually reached** by this approach and **how** did you reach these actors?

m. Could you also tell me to what extent there were **groups** or individuals in the community where it was not possible or where it was very **difficult to involve them**?

Assessment of the approach

a. What **consequences** or **results** have been shown? (e. g. benefits)

b. Were the **results evaluated**? In what way?

Conclusion specific participation of citizens

c. All in all, if you think about the areas: Planning, Implementation and Evaluation: In your opinion, ***where,*** in particular, and ***how*** were users or citizens or their representatives involved?

Is there anything else you would like to tell me about this?

6. General factors/aspects hindering and promoting the strengthening of community participation and community empowerment

a. If you think of **different population groups**, e. g. different age groups, different socio-economic classes, people with certain chronic diseases or with a migration background: Do you see differences if and how these different groups can or want to be involved? Can you give an example?

b. In your opinion, what **factors/aspects hinder or inhibit** the promotion of community participation or empowerment in the Case della Salute?

c. And, conversely, what are some **supporting factors/aspects** that promote community participation or empowerment in the Case della Salute?

d. What **suggestions for improvement** would you make to further support and promote community participation and community empowerment in the Case della Salute?

e. Are there any activities or training to **support professionals** in promoting community participation and community empowerment in the Case della Salute?

7. Future developments

a. Do you know if there are any **plans, strategies or ideas**, which aim to further develop community participation and community empowerment in the context of the Case della Salute? If yes, could you tell me about them?

b. If you think **5-10 years ahead**, what do you think how the Casa della Salute will **develop**?

   a. in terms of community participation and community empowerment
   b. in general

8. Participation in teams of physical distance

a. Thinking about the **physical distancing of people** we are experiencing these days (due to the pandemic), in your opinion, how could community participation and community empowerment be supported in the future in the context of the Case della Salute?

9. Now we talked for quite a while and you told me a lot of things. We come to the end of the interview**. Is there anything else you think is important and want to tell me?**

10. Other interview partners and interesting Case della Salute

a. Do you know of other regional or local experts or Case della Salute that are particularly interesting for our study because they follow approaches that promote community participation and empowerment?

For example: Stakeholders at the level of the Emilia-Romagna Region, stakeholders at the level of municipalities, Case della Salute that have a CdS-board where citizens / their representatives are represented; Case della Salute that have used a Community Lab; Case della Salute with various community projects etc.
