# Supplementary material for: Understandings of community participation and empowerment in primary health care in Emilia-Romagna, Italy: A qualitative interview study with practitioners and stakeholders
Source: PLoS One. 2024 Sep 26;19(9):e0310137. doi: 10.1371/journal.pone.0310137 (PMC11426527; doi:10.1371/journal.pone.0310137)
Supplement: Attachment — Submitted filename: Response-to-Reviewers-CP-CE-Emilia-Romagna.docx [file pone.0310137.s003.docx]

*Revision no 1 of PONE-D-23-42209*

*Title: Understandings of community participation and empowerment in primary health care in Emilia-Romagna, Italy: A qualitative interview study with practitioners and stakeholders*

**Response to Reviewers**

Dear Dr. Prenestini, dear Reviewers,

Thank you very much for considering our article for revision. We did a major revision and gave our best to address all the concerns and questions. We provide below all the answers to your concerns.

Thank you again for the opportunity to resubmit.

Sincerely,

Kerstin Hämel and Daniela Luisi

| Additional Editor Comments:  The paper is really interesting, but some of the reviewers raised concerns about the lack of a theoretical framework. This is the more critical point of the reviews.  Moreover, they required some clarifications about the selection of the sample that, in my opinion, are right.  Therefore, I would ask you to improve the paper with their suggestions and to clarify some of the doubts that they had in reading your study.  At the same time, I think that 19 interviews may be an adequate number and that the choice to select stakeholders within several different institutional levels can be relevant to the study. | Thank you very much for giving us the possibility to improve our manuscript and resubmit it. We understand that some parts need to be clarified further and we did our best to modify and deepen the named aspects and address the raised concerns.  We did major revisions to properly address all of the reviewers’ concerns.  In particular we substantially revised the introduction section to delineate the theoretical framework which builds the basis of our study (see here also answers to reviewers 2 and 3) and enriched our literature review (see answers to review 1 and 3).  We agree that the selection of the sample was not very clearly explained. We now present a much more detailed description to clarify why and how we selected the study participants as well as addressed concerns regarding the seize of the sample. We give detailed answer to this concern in the point-by-point protocol, in particular for reviewer 1 and 3.  We thank you for your comment regarding the number of the interviews. We agree that 19 interviews build a proper basis for our analysis as we purposively selected these persons and stopped recruiting more participants only when data saturation was reached. We explain this in further detail addressing reviewer 1 and 3. |
| --- | --- |
| The final responses of the three reviewers are completely different from each other. Two reviewers raised crucial concerns about the lack of a comprehensive theoretical framework upon which the research is built. | We now added and explained more in detail in the introduction section the theoretical framework we used and build on it also in the discussion (see answers to reviewers 1 and 3). |
| Moreover, it seems that it is not completely clear which is the context of the sample. Have you considered only the stakeholders within only a Local Health Authority, or in a large area with several LHA, or the entire Emilia Romagna Region? In the second or the latter case, are there any differences in the experts' responses due to the different contexts in the Emilia Romagna Region you have taken into account?  In my opinion, the paper is really interesting and useful for its results, but it requires major revisions. Please follow the previous and the other suggestions by the reviewers to improve your research paper | We considered stakeholders as well as practitioners from different provinces of Emilia-Romagna region. We added these aspects related to the sampling in the text, so to make it clearer and more transparent: We described and argued more explicitly the choice of the sampling. We clarified that we were looking for good-practice examples to increase variability and that is why we searched all over the region for CdS/CdC that promote CP and CE (see detailed description in response to reviewer 1 and 3).  Moreover, we highlighted aspects related to the different contexts in the Emilia-Romagna region by adding quotations and descriptions in the text (results section) (see detailed description in response to reviewer 2) and by reflecting on it in the discussion and in the section dedicated to the strengths and limitations of the study. |

| Reviewer’s feedback | Author’s responses with reference to the lines in the document ‘Manuscript’ |
| --- | --- |
| Reviewer #1: |  |
| The topic is relevant and quite interesting indeed. the paper fails to present the literature review on this topic and specifically on that Emilia Romagna case. so it is not clear which is the debate the author(s) want to join and provide a contribution | Thank you very much for your feedback that guided us to substantially revise our manuscript.  We completely revised the introduction section. We explicated the literature review and the theoretical framework our study is built on drawing on theoretical and conceptual literature on primary health care, Community Participation (CP) and Community Empowerment (CE) (see lines 55-108).  Moreover, we present much more literature and details on the case of Emilia- Romagna region in order to highlight the unique situation of this region (see lines 109-118 and 139-154). |
| it is not clear if the 19 professionals are from the same LHA or not. It should because the LHA are very different and CdC are very different within the same Region (e.g. organized differently). | We thank you for raising this important aspect. We did major revisions of the methods chapter to address this and the other information (see following answers) you requested, so to make it clearer and more transparent.  We explain now in greater detail how we have selected the study participants (stakeholders and practitioners) and justify our sampling strategy encompassing professionals from different CdS/CdC from different LHAs (see lines 178-218).  We clarified that we selected CdS/CdC which are managed by different LHAs, because they were identified by literature, by the stakeholders and by other practitioners as good-practice examples for the promotion and development of CP and CE in Emilia-Romagna (see lines 178-184, lines 193-198, lines 199-209).  This focus on good practices made it necessary to include CdS/CdC all over the regional territory to build a robust sample. Moreover, not all CdS/CdS that are managed under the same LHA are fostering CP and CE equally. Therefore, we decided not to take the LHAs as cases, but the good-practice-examples of CdS/CdC (see lines 199-213). |
| The doctor are Gps or not ? if yes how they are selected? | Thank you for requesting more details of the study participants characteristics. We specified the characteristics of the study participants in more detail. We also present in table 1 how many of the physicians were GPs and how many hold another medical specialization. (see lines 230-231). We added the selection criteria and process of the practitioners (which includes the GPs) (see lines 188-198) |
| It is almost impossible to benchmark two different LHA, so the analysis should have been done at regional level only or for a single LHA or otherwise using the same approach used by Longo et al or Compagni et al. | We read the publications of Longo et al. and Compagni et al. and understand your point in comparing different LHAs or the CdS/CdC within a single LHA.  However, please consider the following aspects:  Through literature review and through stakeholders interviews we understood that the CdS/CdC have some autonomy in deciding if and how to foster CP and CE, even if they are under the same LHA. Moreover, the local community (including the administration and municipality) and already established collaborations with the third-sector, as well as the community/population characteristics are also aspects that influence whether CP and CE are fostered within the CdS/CdC or not.  We, therefore, decided to choose the CdS/CdC as cases and not the LHAs. This allowed us to focus on good-practice examples of CdS/CdCs. The fact that they belonged to different LHAs, different provinces and different local contexts (e.g. urban/rural CdS, different local community/ third sector), provided us manifold and rich description as in line with a qualitative approach (see line 199-218). We moreover discussed this aspect in the section strengths and limitations (see lines 637-650)    Sampling and field access are accordingly revised to describe more clearly the reasons for choosing a purposive sample strategy and the CdS/CdC as cases (see above). |
| Reviewer #2: |  |
| Dear authors, I want to express my gratitude for allowing me to engage with your work titled "Understandings of community participation and empowerment in primary health care in Emilia-Romagna, Italy: A qualitative interview study with practitioners and stakeholders."  Your paper is well-written, concise, and engaging. It addresses several pertinent themes such as change management and community health, providing valuable insights. The abstract aptly encapsulates the essence of the article, and the research objective is clearly delineated. The methods employed are appropriate and well described. The results presented are significant, offering insights that practitioners, stakeholders, and policymakers can leverage, particularly regarding the conceptual categorization of community participation and empowerment within outpatient facilities like Case della Salute (CdS), now Case della Comunità (CdC). The discussion and conclusions effectively frame the results, highlighting both the accomplishments of the paper and its limitations. While I believe the paper is well-suited for publication, I have some suggestions for revisions. | We thank you very much for the encouraging feedback. |
| Title and Terminology  The paper lacks an explicit definition of primary care. It's worth considering that, according to both Italian and English terminologies, CdC extends beyond the boundaries of primary care. In many Beveridge systems, primary care typically refers to general practices offering gatekeeping services for adults and children.  However, CdC aims to integrate various services and professional profiles, including specialist physicians, psychologists, obstetricians, etc.  Therefore, I recommend revising the title and terminology throughout the paper to incorporate phrases such as "outpatient (healthcare) services" to better reflect the scope of CdC | We thank you very much for critically reviewing the terminology. In our revision we added, in the introduction, a definition of “*primary health care”* and described more in detail the services of the Italian CdS/CdC. Using the definition of *primary health care* by WHO we understand a comprehensive approach that not only includes general practice, but also different other specialists and services like specialist outpatient care. We believe that this is coherent with your suggestions of not limiting PHC only to general practice as it is often understood in classical Beveridge systems and described in “*primary care”* (see lines 55-62).  We pointed this also out by describing more in detail that the CdC intend to go beyond GP services. We did so by citing relevant scientific Italian literature on this topic and giving examples of the services which are offered in the health centres and professionals working in the CdS/CdC (see line 109-118; lines 126-138). |
| Results While the themes regarding participants' understandings of CP and CE are compelling, there are a few aspects that could enhance the comprehensiveness and soundness of the results you present. Firstly, I suggest indicating the exact number of interviewees who mentioned a specific theme, rather than relying on vague expressions like "According to many interview participants" (line 273). | We intensively discussed if we should quantify the number of persons that mentioned a specific theme or not. As a result, we decided not to follow your suggestion. Our decision was built on the following considerations, that we kindly ask you to consider:  We know that there are some qualitative content analysis approaches that quantify the distribution of answers and indeed, with our language use, we indicated such practices as you pointed out. However, this is not in line with the method we chose (qualitative content analysis according to Kuckartz, 2012/2023).  Therefore we added this aspect an in the methods section, arguing that with our qualitative study design, we want to show which theoretical and practical understandings of CP and CE emerge drawing on good practice examples. A qualitative approach allows us to explore perspectives and experiences of actors in-depth and in detail in specific and complex contexts, so to understand nuances of a phenomenon like community participation and empowerment. The focus lies on the identification of rich and dense categories and codes without weighting one more than another. So, how often an aspect was mentioned or how many people described an aspect is not necessarily to be equated with importance (see lines 167-176).  Moreover, we now changed those parts in the text that were vague due to the expressions “some” and “many”. We highlighted only when it was just one person who reported an opinion/experience. |
| Secondly, providing additional details about the professional profiles of the practitioners or stakeholders, such as whether they are nurses or physicians, would offer further context to the evidence presented. | We specified in table 1 some more characteristics of the practitioners and stakeholders (see lines 230-231). Moreover, in the quotations presented in the results chapter we added the profession of the interviewees involved in CP and CE activities (see for examples lines 311; 319; 344; 352; 360).  However, as we interviewed six stakeholders that are experts in this field, and these might be known nationwide, we have to make sure to protect their privacy and therefore did not specify their profession as they might be recognized from people in the field. |
| Thirdly, the development of CP across CdC experiences appears to be uneven, influenced by various factors including the ideas and values of management and practitioners, as well as the availability of local resources (line 190). While this is a crucial issue, it is somewhat briefly addressed without providing direct quotations. I recommend expanding and elaborating on this aspect. Best regards | We expanded this aspect by describing it in the beginning of the results section more in detail and added quotations to support it (see line 297-319). Moreover, we referred to these results in the discussion, underlining its importance for the understanding of CP and CE (see line 527-536). |
| Reviewer #3: |  |
| Synthesis: The study investigates the perceptions of community participation (CP) and empowerment (CE) among practitioners and stakeholders involved in primary health care (PHC) initiatives within the Emilia-Romagna region. The manuscript offers valuable insights into the varied understandings of CP and CE within the context of PHC, shedding light on the complexities inherent in participatory processes. The methodology employed, including semi-structured interviews and qualitative content analysis, appears appropriate for addressing the research objectives outlined in the abstract. However, I believe that the manuscript requires significant revisions before it can be considered suitable for publication. My major concerns and suggestions for revision are outlined below: | Thank you very much for the constructive feedback. We hope that we could properly address your concerns by the further development of the paper. |
| INTRODUCTION: The introduction of the paper outlines the significance of community participation (CP) and empowerment (CE) in primary healthcare (PHC) within the context of the Emilia-Romagna region in Italy. To further enhance the introduction and clearly articulate the contribution to the international literature, consider expanding on the following points: | We completely revised the introduction chapter and addressed the raised concerns one by one as follows: |
| - The manuscript would benefit from a more explicit engagement with relevant theoretical frameworks or literature on community participation and empowerment. Incorporating theoretical perspectives could enrich the analysis and provide a stronger conceptual foundation for the study. | We now enriched our literature review in the introduction, adding much more literature on the topics of PHC, community participation and empowerment (e.g. drawing on scholars like Kringos and Boerma, WHO, Starfield, Rifkin and colleagues, Laverack, Arnstein and others). Moreover, we presented the theoretical framework on which our study was build (Brunton’s et al. Framework on community participation) (see lines 56-108).  We used the chosen framework and the literature to enhance our analysis and discussion (see lines 566-579) |
| - Emphasize the unique characteristics of the Emilia-Romagna region that make it an important case study for understanding CP and CE in PHC. Highlight any distinctive features of the regional healthcare system.  By addressing these aspects, the introduction will provide a comprehensive overview of its contribution to the international literature on CP, CE, and PHC | In the introduction, we added more literature on particular aspects of the Italian health care system and on the unique characteristic of Emilia-Romagna in the national picture (e.g. long history of collaboration with third sector, participatory approaches), so to highlight its distinctive features, as well as on the CdS/CdC and their particularities in an international panorama (see lines 109-118; lines 119-154). |
| METHOD: - I have some doubts about the size of the participants (N 19). The authors should explain more clearly the percentage of representativeness of the sample. | We understand your concern regarding the representativeness of the sample. However, please consider the following arguments:  In accordance with a qualitative paradigm, the focal point is not on representativeness of the sample, but on the exploration of information-rich cases. A qualitative approach allows us to explore perspectives and experiences of actors in-depth and in detail in specific and complex contexts, so to understand nuances of a phenomenon like community participation and empowerment. The focus lies on the identification of dense categories and codes without weighting one more than another. So, how often an aspect was mentioned or how many participants described an aspect is not necessarily to be equated with importance.  However, in the methods section, we further explicated our sampling strategy and the selection of the cases (e.g. selection of good-practice examples, different CdS/CdC from different provinces in Emilia-Romagna etc.) (see lines 177-218). |
| - Highlight the methodological rigor of your study, particularly in terms of data collection, analysis, and interpretation. Discuss how the use of qualitative content analysis and semi-structured interviews enhances the depth and richness of the findings, adding credibility to the study's contribution to the literature. | We added criteria to ensure rigor/trustworthiness of qualitative research for data collection, analysis, and interpretation, referring to international literature on quality criteria for qualitative research (e.g. O’Brien et al. (2014) Standards for Reporting Qualitative Research: A Synthesis of Recommendations; Connelly (2016) Trustworthiness in Qualitative Research (see lines 273-276, lines 286-294). |
| DISCUSSION: Discuss how insights gained from studying CP and CE in Emilia-Romagna contribute to broader international discussions on PHC.  Consider contrasting the findings with existing literature from other regions or countries to identify similarities, differences, and potential transferability of lessons learned. | We now discussed the results with more international literature on CP and CE and highlighted contribution of the findings to a broader international discussion (see lines 511-517; lines 527-536; lines 537-543; 566-590). We formulated lessons learned that can be useful for Italian national and international health centre/PHC contexts (see lines 612-627). |
